# Genome-wide association study identifies novel risk loci for apical periodontitis

**DOI:** 10.21203/rs.3.rs-2515434/v1

**Published:** 2023-01-26

**Authors:** L.E. Petty, R. Silva, L. Chaves de Souza, A.R. Vieira, D.M. Shaw, J.E. Below, Ariadne Letra

**Affiliations:** Vanderbilt University Medical Center; University of Pittsburgh School of Dental Medicine; University of Texas Health Science Center at Houston School of Dentistry: The University of Texas Health Science Center at Houston School of Dentistry; University of Pittsburgh School of Dental Medicine; Vanderbilt University Medical Center; Vanderbilt University Medical Center; University of Pittsburgh School of Dental Medicine

**Keywords:** gene association, pulp necrosis, periapical lesion, susceptibility

## Abstract

Apical periodontitis (AP) is a common consequence of root canal infection leading to periapical bone resorption. Microbial and host genetic factors, and their interactions, have been shown to play a role in AP development and progression. Variations in a few genes have been reported in association with AP however, the lack of genome-wide studies has hindered progress in understanding the mechanisms involved in AP Here, we report the first genome-wide association study of AP in a well-characterized population. Male and female adults (n=932) presenting with deep caries with AP (cases) or without AP (controls) were included. Genotyping was performed using the Illumina Expanded Multi-Ethnic Genotyping Array. Single-variant association testing was performed adjusting for sex and five principal components. Subphenotype association testing, analyses of genetically regulated gene expression, polygenic risk score and phenome-wide association (PheWAS) analyses were also performed. Eight loci reached near-genome-wide significant association with AP (p < 5 × 10–6); gene-focused analyses replicated three previously reported associations (p < 8.9 × 10–5). Sex-specific and subphenotype analyses revealed additional significant associations with variants genome-wide. Functionally oriented gene-based analyses revealed eight genes significantly associated with AP (p < 5 × 10–5), and PheWAS analysis revealed 33 phecodes associated with AP risk score (p < 3.08 × 10–5). This study identified novel genes/loci contributing to AP and revealed specific contributions to AP risk in males and females. Importantly, we identified additional systemic conditions significantly associated with AP risk. Our findings provide strong evidence for host-mediated effects on AP susceptibility.

## Introduction

Apical periodontitis (AP) is a common inflammatory disease that results from the progression of microorganisms within an infected/necrotic root canal system. The localized infection activates the local host immunoinflammatory response, triggering a cascade of events with recruitment of immune cells, release of inflammatory mediators, local inflammation, hard tissue breakdown, and eventual formation of a periapical lesion (Graves et al., 2011; Menezes-Silva et al., 2012; Stashenko et al., 1998; Stashenko et al., 1992).

AP affects approximately 25% of the general population worldwide and imposes major health and financial burdens to affected individuals and society, with billions of dollars spent on treatment costs every year in the US (Mehrazarin et al., 2017). Between 30% to 96% of all root treatments are successful, depending on a number of variables, including the presence or absence of AP (Mehrazarin et al., 2017). If left untreated, however, AP can lead to additional local and/or systemic consequences, including life-threatening conditions, such as cellulitis and angina (Moghimi et al., 2013).

Characterizing the etiology of AP is critical to improve understanding of disease pathogenesis and inform future prevention and treatment strategies (Cavalla et al., 2021). Historically, AP was considered a strictly infectious condition of complex polymicrobial nature, hence the focus of the majority of studies on identifying microbial species associated with the condition, and on antimicrobial substances as adjuvants in endodontic therapy (Siqueira & Rocas, 2021). More recently, studies in humans and animal models have implicated host factors as critical players in AP susceptibility, with host-pathogen interactions suggested to influence disease development and progression (Cavalla et al., 2021; Dill et al., 2015; Maheshwari et al., 2016; Menezes-Silva et al., 2012).

Host factors such as genetic polymorphisms may affect host physiology, but they are also likely to affect the interactions between pathogen and host. Pathogens use host genes/proteins during their life cycles, and the host deploys many immune, inflammatory and defensive genes against invading pathogens (Carter et al., 2017). Therefore, disease susceptibility genes relevant to pathogens may divert the effects of the pathogen toward or away from their functional pathways, thus influencing diverse diseases in a manner that depends on host genetics (Carter, 2013a, 2013b). Evidence for host genetics as a contributor to AP susceptibility comes from clinical observations in patients who have deep caries (in close proximity to the pulp or already invading the pulp space) and yet resist the development of AP in contrast to patients with similar deep caries lesions who develop AP quite readily (Menezes-Silva et al., 2012). These findings indicate that patients may present with similar affection status (deep caries), but distinct clinical diagnosis and periapical presentations (AP versus no AP). Moreover, variants in genes belonging to immuno-inflammatory pathways have been reported in association with AP subphenotypes (Aminoshariae & Kulild, 2015; Dill et al., 2015; Maheshwari et al., 2016; Menezes-Silva et al., 2012; Morsani et al., 2011; Trombone et al., 2016). While these studies have strengthened the evidence for host-mediated modulatory effects on AP much remains to be learned about the molecular players and mechanisms involved in AP pathogenesis. Importantly, the presence of AP has been associated with systemic conditions such as diabetes and cardiovascular disease (Aminoshariae et al., 2017; Jakovljevic et al., 2020; Liljestrand et al., 2016; Messing et al., 2019). These findings suggest that co-occurrence of AP and systemic conditions may be mediated by host genetics, potentially contributing to shared predisposition; this further highlights the critical need for dissecting the genetic etiology of AP (Messing et al., 2019).

In this study, we performed the first genome-wide association study of AP to identify novel genes and loci contributing to AP susceptibility. Genetically regulated gene expression, polygenic risk score, and phenome-wide association (PheWAS) analyses were also performed to understand the functional consequences of AP-associated loci. Our findings reveal novel genes and loci contributing to AP and additional systemic conditions significantly associated with AP risk.

## Materials And Methods

### Ethics Statement

This study was approved by the Institutional Review Boards at UTHealth-Houston and University of Pittsburgh. The Strengthening the Reporting of Observational Studies in Epidemiology (STROBE) guidelines were used for reporting this observational human research study. Written informed consent was obtained from all participants.

### Subject population

Male and female adults (n=932; 395 males, 537 females, ≥ 18 years old) enrolled for treatment at the UTHealth School of Dentistry Endodontic clinics or at the University of Pittsburgh School of Dental Medicine were invited to participate (Supplementary Table 1). Individuals recruited at the University of Pittsburgh did so via the DNA Registry and Dental Repository (DRDR). For each individual, basic demographic, medical and dental record information were assessed using structured questionnaires and/or electronic health record (EHR) data. Exclusion criteria included patients with previous endodontic treatment, with medical conditions requiring the use of systemic modifiers of bone metabolism or other assisted drug therapy (i.e., systemic antibiotics, anti-inflammatories, hormone therapy) during the six months preceding entry into the study, smokers, and patients with preexisting conditions such as periodontal disease, as these may function as modifiers that complicate the relationship between genetic risk factors and disease (Garlet et al., 2012).

### Clinical phenotyping and AP assessment

Diagnostic procedures for AP followed standard-of-care criteria established by the American Association of Endodontists (Glickman, 2009). Clinical and radiographic examinations were performed to detect the presence of caries and/or pulpal/periapical pathologies (Fava & Dummer, 1997). Thermal and electric pulp sensibility tests were performed to determine pulpal diagnosis; palpation and percussion tests were performed to determine periapical diagnosis (Alghaithy & Qualtrough, 2017; Dill et al., 2015; Maheshwari et al., 2016; Menezes-Silva et al., 2012). An AP lesion was characterized radiographically as a rarefaction lesion with the disappearance of the periodontal ligament space and discontinuity of the lamina dura (Hans-Göran Gröndahl & Huumonen, 2004). Individuals considered cases had deep carious lesions (involving at least 2/3 of the dentin depth) for which diagnostic testing confirmed a pulpal diagnosis of symptomatic irreversible pulpitis or pulp necrosis and a periapical diagnosis of asymptomatic or symptomatic AP acute apical abscess, or chronic apical abscess, and radiographic evidence of periapical lesion >2mm. Individuals considered controls had deep carious lesions (involving at least 2/3 of the dentin depth), for which diagnostic testing confirmed a pulpal diagnosis of vital pulp tissues and/or with reversible pulpitis, and a periapical diagnosis of normal apical tissues (i.e., no AP lesion).

### Genotyping

Genomic DNA was extracted from saliva samples using established protocols (Letra et al., 2012). DNA purity was assessed by measuring the A260/A280 ratio using NanoDrop (Thermofisher), and only DNA samples with absorbance values >1.7 were considered acceptable. Samples were genotyped using the Expanded Multi-Ethnic Genotyping Array (MEGA^EX^, Illumina, San Diego, CA), which contains > 2 million markers covering exonic, promoter, indel, missense, nonsense, and UTR variants, with >270,000 variants reflecting Gene Ontology conditions, >11,000 variants from published GWAS, and >23,000 variants included for functional immunological, oncological, ancestry, forensic and common and rare disease research applications. This array is also optimized for high imputation accuracy for minor allele frequencies >0.5% across all continental populations, enabling us to improve power and accuracy in our multi-ancestry study.

### Quality control and imputation

Variants with minor allele frequency < 1 %, missing in > 5% of samples, or deviation from Hardy-Weinberg equilibrium (HWE) at *p* < 1×10^−5^, and samples missing > 10% of variants, with outlying heterozygosity, or mismatch of genetic and expected sex were removed prior to imputation. This resulted in a total of 879 individuals, 333 AP cases and 546 controls. Principal components were generated using PLINK 1.9 (Chang et al., 2015) to capture population structure. Following quality control, genotype data were aligned to TOPMed freeze 8 reference data using the TOPMed Imputation Server, with haplotype phasing performed using Eagle and imputation performed using Minimac4 (Das et al., 2016; Fuchsberger et al., 2015). Variants with allele frequency differences > 20% or allele inconsistencies with the reference panel were removed.

### Genome-wide single variant association testing

Single variant association analyses were performed using SAIGE (Zhou et al., 2018), which utilizes a generalized mixed model to account for relatedness and a saddlepoint approximation to allow for imbalance in case and control sample sizes. The imputed data was filtered to retain variants with minor allele frequency > 1 % and imputation R^2^ > 0.5. Genome-wide association analyses for apical periodontitis were conducted, adjusting for five principal components, and i) adjusting for sex and ii) in sex-specific male and female analyses. Additional exploratory analyses of AP subphenotypes considering the presence or absence of pulpal and periapical pain were also conducted, adjusting for sex and five principal components. A conventional *p* < 5 × 10^−8^ threshold was used to denote genome-wide significance; *p* < 5 × 10^−6^ was used to identify variants suggestively associated with the phenotype. The variant with the lowest *p* value within each locus was selected as the sentinel variant. Each sentinel variant was then annotated using ANNOVAR to determine its position relative to the nearest gene(s) (Wang et al., 2010).

We also performed a candidate-gene based association analysis considering genes previously associated with AP or disease-relevant genes suggested by animal model studies, to validate findings (Aminoshariae & Kulild, 2015; Cavalla et al., 2021; Dill et al., 2015; Maheshwari et al., 2016; Menezes-Silva et al., 2012; Morsani et al., 2011; Rocas et al., 2014). Bonferroni correction was applied (0.05/31 tests) and *p* values < 0.001 were considered statistically significant.

### Power calculations

We calculated power for our single variant GWAS analysis using GAS Power Calculator (Johnson, 2017)

Assuming 330 cases and 550 controls, a simple logistic regression model with additive effect, and a prevalence of 0.5, we are 80% powered at genome-wide significance (a=5×10^−8^) to detect a common variant (MAF = 0.25) with an OR of 1.42, or a less common variant (MAF = 0.1) with an OR of 1.60.

### PrediXcan analysis

Genetically regulated expression (GReX) levels were estimated using PrediXcan (Gamazon et al., 2015), which employs transcriptome prediction models derived from reference expression and genotype data. Using Joint-Tissue Imputation tissue-specific expression models (Zhou et al., 2020) for 49 tissues built in the Genotype-Tissue Expression (GTEx) project (Consortium, 2020) reference data, gene expression was imputed for each gene for all available tissues. The imputed expression values were tested for association with AP using a logistic regression model, adjusting for sex and five principal components. Then, to assess for causality of the genes identified using PrediXcan, we used the Mendelian randomization approach described in (Zhou et al., 2020). We created LD scores for all variants in the single variant GWAS results using GCTA v1.93.2 (LD window size = 1000 kb, LD r^2^ threshold = 0.01) (Yang et al., 2011). For expression quantitative trait locus (eQTL) data, we used publicly available Genotype Tissue Expression Project v8 “eQTL Tissue-Specific All SNP Gene Associations” data (https://www.gtexportal.org/home/datasets), retaining for each gene-tissue pair, variants associated with gene expression with *P* value < 0.05 and all variants present in the JTI model.

### Polygenic risk score

We used the summary statistics resulting from our AP GWAS to develop a polygenic risk score (PRS) model using data from 9,221,680 autosomal variants. The PRS model was developed using the PRScs Python tool (https://github.com/getian107/PRScs), which uses an external linkage disequilibrium (LD) reference (the 1000 Genomes Project phase 3 European superpopulation reference panel) and continuous shrinkage model priors on SNP effect size to calculate SNP weights from the AP summary statistics. Our global shrinkage parameter (phi) was set to “auto”.

### Polygenic risk score application

We applied our AP PRS to genetic data in the Vanderbilt University Medical Center biobank with linked electronic health records, BioVU. All BioVU subjects were genotyped on Illumina’s MEGA^ex^ Array. Quality control was performed using PLINK v.1.9. All indels and duplicate variants, variants with call rates < 98% and samples with call rates < 97% were removed. Principal components were generated for ancestry classification. Samples were separated into five ancestry groups based on principal components: European, African, East Asian, Hispanic/Latino, and South Asian. Additional ancestry-specific quality control steps were performed, removing all variants with a minor allele frequency < 1 % and variants missing in > 5% of samples. Variants that deviated from Hardy-Weinberg equilibrium were also removed (*p* < 1 × 10^−10^). Haplotype phasing and imputation to TOPMed freeze 8 were performed using Eagle and Minimac4 on the TOPMed Imputation Server (Das et al., 2016; Fuchsberger et al., 2015).

For application of our AP PRS, we restricted to individuals of European ancestry, which comprise the largest BioVU ancestry group. Individual polygenic scores were calculated using PLINK v.1.9. We tested two AP case definitions for BioVU subjects, based on International Classification of Disease (ICD-10) codes. A broader AP case definition included all individuals with at least one instance of an ICD code under the category of “Disease of pulp and periapical tissues” (K04, K04.*) as a case. A narrower case definition included only individuals with at least one instance of ICD codes for pulpitis (K04.0), reversible pulpitis (K04.01), irreversible pulpitis (K04.02), acute apical periodontitis (K04.3), or chronic apical periodontitis (K04.4) as a case. We used a two-sample t-test to compare the overall score distribution between AP cases and controls.

### Phenome-wide association analysis (PheWAS)

We used the PheWAS R package (Carroll et al., 2014) to run a logistic regression analysis, testing association of each BioVU health record phecode against the AP polygenic risk scores calculated within BioVU. The analysis was restricted to phecodes present in at least 30 subject record sets. The logistic regression model was adjusted for age, sex, and record length. Record length was defined as each subject’s number of unique clinical visits. We corrected for multiple testing by applying a Bonferroni correction for 1,620 phecodes.

## Results

Of the 932 study subjects, 53 were excluded during quality control procedures, and 879 individuals (333 cases and 546 controls) were included in the analyses (Supplementary Table 1). A total of 1,736,793 variants passed post-imputation quality control procedures and were included in the single-variant GWAS.

### Single variant GWAS findings

**Figure 1:**
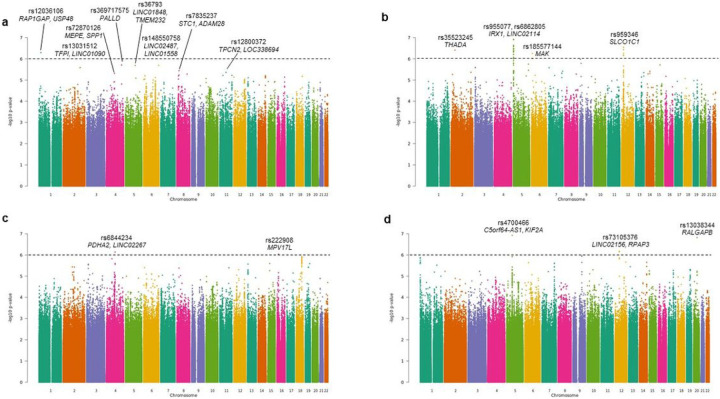
Manhattan plot showing the results of our genome-wide association study of AP. **a**, AP vs. controls; **b**, AP subphenotypes vs. controls; **c** and **d**; sex-specific analysis in females (C) and males (D). Each individual dot represents one SNP. Each point represents a tested genetic variant, in order by location. P-values shown on -base-10 logarithmic scale. The dashed line represents the *P*_threshold_ = 5 × 10^−6^

To assess for possible genomic inflation, we calculated the genomic control lambda for our single variant GWAS findings;, indicating no inflation. No associations were observed reaching formal genome-wide significant threshold levels (*p*< 5 × 10^−8^), however eight loci showed near-genome-wide significant association signals for AP (*p*< 5 × 10^−6^). The strongest associations were observed for chr.1/rs12036106 (*p*=5.07 × 10^−7^), chr.4/rs369717575 and rs72870126 (*p*=1.96 × 10^−6^ and 4.8 × 10^−6^, respectively), chr.6/rs148550758 (*p*=2.04 × 10^−6^), chr.5/rs36793 (*p*=2.07 × 10^−6^), and chr.2/rs1303151 (*p*=2.6 × 10^−6^), chr.8/rs7835237 (*p*=3.62 × 10^−6^), and chr.11/rs12800372 (*p*=4.34 × 10^−6^) (Fig. 1 a, Table 1).

When stratifying AP subphenotypes by the presence (symptomatic apical periodontitis, acute apical abscess) or absence (chronic apical abscess, asymptomatic apical periodontitis) of associated pain based on endodontic diagnostic criteria, suggestively significant associations were found for symptomatic AP with 15 variants, including top signals on chr.5/rs955077 (*p*=1.22 × 10^−7^), chr.12/rs959346 (*p* = 2.87 × 10^−7^), chr.2/rs35523245 (*p* = 3.89 × 10^−7^) and chr.6/rs185577144 (*p* = 5.59 × 10^−7^) (Fig. 1 b, Table 2).

Sex-specific single-variant association analyses revealed numerous loci preferentially associated with AP in females or males. Female-specific associations were seen with chr.4/rs6844234 (*p* = 7.32 × 10^−7^) and chr. 16/rs222908 (*p* = 9.24 × 10^−7^). Male-specific associations were observed with chr.5/rs rs4700466 (*p* = 1.19 × 10^−7^), chr.20/rs13038344 (*p* = 1.52 × 10^−7^), and chr.12/ rs73105376 (*p* = 6.65 × 10^−7^), whereas (Table 3, and Fig. 1c, 1d).

We also performed focused candidate gene-based association analyses considering genes previously suggested as potentially associated with AP and additional disease-relevant genes suggested by animal models studies of AP (Aminoshariae & Kulild, 2015; Cavalla et al., 2021; de Souza et al., 2019; Dill et al., 2015; Maheshwari et al., 2016; Menezes-Silva et al., 2012; Messing et al., 2019; Morsani et al., 2011). The strongest suggestive associations point to variants in the *IL4*, *TNF*, and *TAC1* loci (*p* < 8.9 × 10^−5^). Variants in the *CRP*, *CCL2*, *CCL8*, *NOS1*, *NOS2*, *NOS3*, *IFNG*, *MMPs-2*,*3*, *7*, *8*, *9*, *TIMP2*, *TLR4*, *PTGER2*, and *NFKB1* loci were also suggestively associated with AP (*p* < 9.4 × 10^−4^) (Supplementary Table 2).

### PrediXcan findings

In our functionally oriented gene-based analyses using PrediXcan, of the 16,709 total genes tested, 8 genes were significantly associated with AP (*p* < 5 × 10^−5^). Genetically regulated expression of *GATC* was associated with AP in five tissues (adrenal gland, one brain tissue, two cell lines, and kidney cortex), while other associated genes, *AC010173*.*1*, *ZNF750*, *POP5*, *ERICH3*, *COX6A1*, *SBSN*, and *AC078788*.*1*, had suggestively significant signals in only one tissue (Appendix Table 2). *GATC*, *POP5*, and *COX6A1* are all in a small region on chromosome 12, suggesting that linkage disequilibrium among variants in the PrediXcan models for these genes, or co-regulation of the genes may be driving association for some of these genes. Mendelian randomization analyses find a significant causal effect of only *GATC* predicted expression on trait.

### Polygenic risk and AP

**Figure 2:**
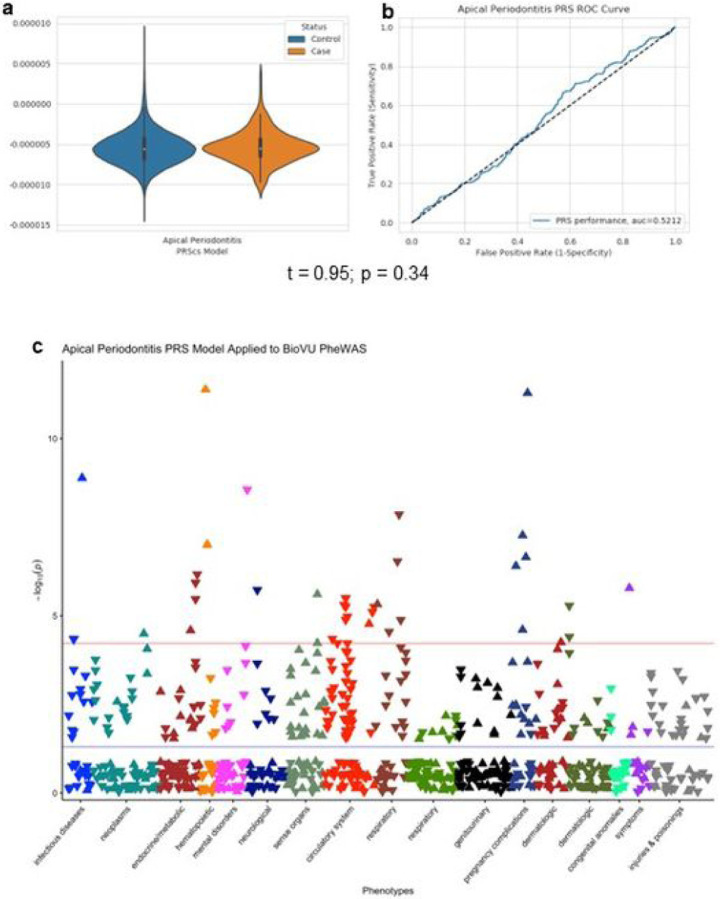
Evaluation of AP polygenic risk score (PRS) and PheWAS of AP. PRS created in the present GWAS population and tested in the BioVU biobank, comparing individuals with AP-related ICD codes to those without the codes. **a,** Violin plot showing distribution of PRS in BioVU AP-related cases compared to controls; **b,** Receiver operating curve showing the ICD codes used: K04 Diseases of pulp and periapical tissues; K04.0 Pulpitis; K04.01 Reversible pulpitis; K04.02 Irreversible pulpitis; K04.1 Pulp degeneration; K04.2 Abnormal hard tissue formation in pulp; K04.3 Acute apical periodontitis of pulpal origin; K04.4 Chronic apical periodontitis; K04.5 Periapical abscess with sinus; K04.6 Periapical abscess without sinus; K04.7 Radicular cyst; K04.90 Unspecified diseases of pulp and periapical tissues; K04.99 Other diseases of pulp and periapical tissues; **c,** Results of phenome-wide association analysis of AP PRS in BioVU. Each point represents a single phenotype, with phenotypes grouped into categories of similar phenotypes; the direction of the triangle indicates the direction of effect

We created a polygenic risk score from our AP GWAS summary statistics and applied it to an independent biobank with linked electronic health records, BioVU (https://victr.vumc.org/), where we tested association of AP risk score with two AP phenotype definitions based on International Classification of Disease (ICD-10) codes. Using a broader AP case definition (i.e., all individuals with at least one instance of an ICD code under the category of “Disease of pulp and periapical tissues” [K04, K04.*]), we identified 394 cases and 72,204 controls in BioVU. We found a higher risk score in cases compared to controls, albeit not statistically significant (two-sample *t* test, *p* = 0.34) (Fig. 2a). The area under the receiver operating characteristics curve (ROC) was 0.52, indicating minimal separation of BioVU AP cases from controls (Fig. 2b).

We also performed a sensitivity analysis using a narrower AP case definition (i.e., including only individuals with at least one instance of ICD codes for pulpitis [K04.0], reversible pulpitis [K04.01], irreversible pulpitis [K04.02], acute apical periodontitis [K04.3], or chronic apical periodontitis [K04.4]), including 39 cases and 72,559 controls in BioVU. We observed slightly less separation in the PRS between cases and controls, though we note that power in the narrower case definition is reduced due to the lower number of cases (two-sample *t* test, *p* = 0.91, ROC = 0.52) (Supplementary Figure 1).

### Phenome-wide association analysis (PheWAS)

Although we did not find evidence of genome-wide significant difference in AP PRS in BioVU, we tested our AP risk score for association with 1,620 other phecodes in BioVU. Using Bonferroni correction, a phenome-wide significance threshold of *p* < 3.08 × 10^−5^ was obtained, resulting in 33 phecodes significantly associated with AP risk score. Among the associated phecodes, nine are related to a circulatory system pathway, five to endocrine/metabolic pathways, and four to hematopoietic pathway, among others (Fig. 2c, Supplementary Table 3).

## Discussion

Here, we report the results of the first GWAS of AP and reveal novel genes and loci contributing to AP risk. Our results show 24 loci reaching neargenome-wide significant association signals, and eight variants associated with higher risk of AP. Consistent with most GWAS studies, most of the associated variants are located in noncoding regions of the genome and may act as proxies of causal variants which are often located in regulatory regions that modulate gene expression (Wang et al., 2010).

In the genome-wide analyses, the strongest associated variant is located close to RAP1 GTPase activating protein *(RAPTGAP)*, a negative regulator of Ras-associated protein1 *(RAP1)*. *RAP1* plays a major role in normal and inflammatory process such as macrophage phagocytosis, chemokine-induced adhesion and leukocyte migration, lymphocyte and dendritic cell homing, as well as adhesion to extracellular proteins such as fibronectin, fibrinogen, collagen, and laminin (Retta et al., 2006). By inhibiting *RAP1* function, *RAPT GAP* appears to contribute to severe impairment of macrophages to ingest opsonized particles (Maffei et al., 2013). The second highest association was noted with a variant in *PALLD* (Palladin, cytoskeletal associated protein), which plays a central role in promoting cell motility. Increased palladin expression and cell migration were observed in wounded tissue, suggesting a conserved function for *PALLD* in post-injury tissue remodeling events (Goicoechea et al., 2008). Of the remaining significantly associated variants, those located within *SPP1* and *MEPE* and those nearby long non-coding RNAs (lncRNAs) are also noteworthy. *SPP1* (secreted phosphoprotein 1) facilitates the attachment of osteoclasts to the mineralized bone matrix and displays high-affinity binding to hydroxyapatite; it also acts as a cytokine that upregulates the expression of IFNG and IL12. *MEPE* (matrix extracellular phosphoglycoprotein) encodes a secreted calcium-binding phosphoprotein that belongs to the SIBLING family of proteins and plays an important role during early odontoblastic differentiation and late dentin mineralization. Long non-coding RNAs (lncRNAs) are emerging as novel promising biomarkers for diagnosis and prognosis of inflammatory conditions such as cancer and cardiovascular disease and have recently been reported as novel players in oral inflammatory disorders including oral squamous cell carcinoma (Magagula et al., 2017; Zhang et al., 2020). Although the expression and function of lncRNAs in AP remain to be elucidated, our results provide new insight into additional regulatory mechanisms in AP pathogenesis.

When stratifying AP into symptomatic and asymptomatic subphenotypes, significant associations were noted with 15 variants suggesting that the individual response to pain in cases of AP may have a genetically linked component. Additional gene-based association analyses revealed novel susceptibility genes in known and new signaling pathways and replicated the association of previously reported genes with AP Most of the associated genes belong to pathways involved in immune-regulation, bone metabolism and peripheral pain. The top associated gene, *IL4*, has been suggested as likely playing a protective role in AP-induced bone resorption due to its antiosteoclastogenic action, as shown in *IL4*-null mice, and faster AP progression (Freire et al., 2021). *TNF* expression in AP tissues and its role in NFkB-mediated bone resorption is also well documented (Alvares et al., 2018), whereas *TAC1*, which encodes products of the tachykinin peptide hormone family (e.g., substance P neurokinin A, neuropeptide K and gamma), is also a plausible player in AP pathogenesis, given its expression during tooth development and role as a neurotransmitter and vasodilator of relevance to the dental pulp (Weil et al., 1995).

Evidence from epidemiological and animal model studies support sex-based differences in predisposition to AP disease as well as response to treatment (Valerio and Kirkwood 2018). In this study, sex-specific single-variant association analyses revealed significant and non-overlapping associations in males and females. The associated genes are dispersed throughout the genome and have not yet been reported to be involved in AP although this merely reflects a limitation of publicly available genome-wide expression data generated from oral/dental tissues. These findings are intriguing and provide evidence of sex bias in predisposition to AP and further corroborate studies in animal models which clearly show sexual dimorphism in periapical inflammation and bone loss (McAbee et al. 2012).

Integrating GWAS and expression quantitative trait loci (eQTL) data is a powerful analytical framework to predict potential susceptibility genes for complex diseases (Li et al., 2022). Here, we utilized PrediXcan to estimate genetically regulated expression and test for association with AP genome wide. Predicted expression of eight genes was significantly associated with AP; in particular, *GATC*, *COX6A1*, and *POP5*expression was noted in brain or pituitary gland; *GATC* expression was predicted to be causal for trait based on Mendelian randomization. Of note, while tissue types in the GTEx project do not include oral/dental tissues (exception for minor salivary gland), gene expression is highly correlated across tissue types for many genes, meaning that even tissues without a clear link to AP can serve as important source for discovery, as gene expression may be very similar in AP-relevant tissues.

AP is a polygenic disorder and therefore association with a single genetic variant is not enough to assess disease risk (de Souza et al., 2019). Instead, the combined effect of a set of variants is necessary to obtain a measure of genetic risk. Though power is often a limitation of sample size, we created a polygenic risk score based on our GWAS findings and applied it to an independent biobank dataset where we tested the association of AP risk scores with AP phenotypes based on ICD codes.

A non-significant difference was found in cases compared to controls; we note that prevalence of AP in BioVU is substantially lower than prevalence estimates elsewhere (Mehrazarin et al., 2017), indicating that AP is likely underreported in the biobank, which likely impacts our power to test our AP risk score in this dataset. Because of these limitations, we note that our PheWAS results should be interpreted cautiously, however, AP risk scores were significantly associated with the risk of 33 phecodes related to circulatory system, endocrine/metabolic, and hematopoietic pathways. These findings reinforce the hypothesis that an individual’s AP risk may be modified based on the presence of other systemic health/disease markers in the background.

Indeed, increasing evidence has shown the association of AP with systemic conditions including diabetes and cardiovascular disease (CVD) (Aminoshariae et al., 2017; Jakovljevic et al., 2020; Liljestrand et al., 2016). Most of the studies, however, reflect epidemiological associations that do not directly address the underlying biological mechanisms potentially contributing to a shared genetic etiology. In a recent prior study, we showed that AP was significantly associated with CVD and related risk factors, particularly hypertension. Further, we showed that a variant in *KCNK3* (potassium two-pore domain channel subfamily k member 3), associated with pulmonary arterial hypertension (Ma et al., 2013), was also significantly associated with AP (Messing et al., 2019). Corroborating these findings, one of the top genes associated with AP identified in this study, *PALLD*, has been previously reported to have variants associated with increased risk of pancreatic cancer type 1 and myocardial infarction (Pogue-Geile et al., 2006). These findings support the potential for a shared genetic predisposition between AP and systemic conditions and further highlight the critical need for dissecting the genetic etiology of AP (Messing et al., 2019).

Our study has some limitations, including that the data was generated from a single dataset of limited size and no replication data is currently available. Nonetheless, our subject population consists of a well-characterized, multi-ethnic sample, including individuals with European, Hispanic/Latino, admixed African, and Asian ancestry. Although population stratification is a common source of potential confounding in genetic studies, we utilized a genotyping array that is well-suited for capturing variants relevant to multi-ethnic study populations and employed stringent analytical methods that correct for population stratification, thereby increasing confidence in our results. Additionally, our data also enabled validation of the association between AP and known candidate genes/pathways (e.g., *ILs*, *MMPs*, *TNF*, *NOS*, *NFKB1*, and *CCL* genes) previously reported in different populations.

## Conclusion

Our study identified novel genes/loci that may contribute to AP phenotypes and strengthened the evidence of sexual dimorphism influencing AP risk. Despite low power to develop a PRS for AP risk, additional systemic conditions were found to be associated with AP genetic risk. Identifying independent phenotype-associated genes with high reliability remains challenging, especially for complex diseases. Overall, our findings provide evidence for host-mediated genetic effects on AP susceptibility.

## Figures and Tables

**Table 1.** Top genes/loci associated with apical periodontitis compared with controls.

Chr	Variant ID^a^	Location	Gene(s)^a^	Alleles (1/2)	AF Allele 2	r^2^^b^	AF cases	AF controls	HWE *P* value controls	P value^c^
1	rs12036106	intergenic	*RAP1GAP* (dist=2988), *USP48* (dist=5954)	C/T	0.14	0.95	0.20	0.11	0.52	**5.07 × 10** ^**−7**^
2	rs13031512	intergenic	*TFPI* (dist=251485), *LINC01090* (dist=229606)	C/A	0.11	1.00	0.06	0.14	0.72	**2.60 × 10** ^**−6**^
4	rs72870126	intergenic	*MEPE* (dist=91351), *SPP1* (dist=37483)	A/G	0.16	0.99	0.12	0.19	0.58	**4.80 × 10** ^**−6**^
4	rs369717575	intronic	*PALLD*	T/TA	0.02	0.88	0.03	0.01	1.00	**1.96 × 10** ^**−6**^
5	rs36793	intergenic	*LINC01848* (dist=107933), *TMEM232* (dist=426660)	C/T	0.94	1.00	0.91	0.96	1.00	**2.07 × 10** ^**−6**^
5	rs116632893	intergenic	*COMMD10* (dist=72480), *LOC101927190* (dist=1646)	C/T	0.01	0.81	0.02	0.00	1.00	8.14 × 10^−6^
6	rs139258898	intronic	*C6orf201*	AT/A	0.03	0.99	0.06	0.01	1.00	9.41 × 10^−6^
6	rs74291093	intergenic	*DTNBP1* (dist=145769), *MYLIP* (dist=320259)	C/T	0.04	1.00	0.01	0.06	0.71	6.31 × 10^−6^
6	rs11751513	intronic	*ATG5*	C/A	0.02	0.92	0.04	0.01	1.00	9.99 × 10^−6^
6	rs2749929	intergenic	*AKAP7* (dist=255142), *ARG1* (dist=34552)	T/C	0.25	0.98	0.33	0.20	0.23	7.61 × 10^−6^
6	rs148550758	intergenic	*LINC02487* (dist=40091), *LINC01558* (dist=60661)	T/C	0.02	0.95	0.05	0.01	1.00	**2.04 × 10** ^**−6**^
8	rs139604353	intronic	*SLC7A2*	T/C	0.04	0.99	0.02	0.06	0.26	6.41 × 10^−6^
8	rs7835237	intergenic	*STC1* (dist=164741), *ADAM28* (dist=274493)	C/G	0.03	1.00	0.05	0.01	1.00	**3.62 × 10** ^**−6**^
8	rs73702527	intergenic	*CSMD3* (dist=738347), *TRPS1* (dist=1233210)	A/T	0.24	0.95	0.31	0.21	0.29	5.26 × 10^−6^
10	rs868304463	intronic	*ADARB2*	G/A	0.05	0.94	0.07	0.04	1.00	8.41 × 10^−6^
11	rs78503045	intergenic	*ELF5* (dist=12270), *EHF* (dist=95029)	A/C	0.02	1.00	0.05	0.01	1.00	6.06 × 10^−6^
11	rs12800372	intergenic	*TPCN2* (dist=13750), *LOC338694* (dist=42881)	G/C	0.25	0.92	0.18	0.29	0.30	**4.34 × 10** ^**−6**^
11	rs59613820	intergenic	*B3GAT1-DT* (dist=159874), *LINC02714* (dist=70410)	G/A	0.07	0.99	0.10	0.06	0.41	6.06 × 10^−6^
12	rs886538	intergenic	*LOC107984507* (dist=22215), *FBXL14* (dist=11586)	G/A	0.96	0.99	0.93	0.97	1.00	7.25 × 10^−6^
12	rs74920677	intronic	*ORAI1*	G/A	0.01	0.98	0.00	0.02	0.15	9.16 × 10^−6^
13	rs1924782	intronic	*ATP8A2*	A/G	0.87	0.93	0.82	0.91	0.10	7.83 × 10^−6^
14	rs2766689	intergenic	*DEGS2* (dist=53329), *YY1* (dist=26170)	G/A	0.02	0.98	0.05	0.01	1.00	6.21 × 10^−6^
16	rs11862628	intronic	*NECAB2*	G/A	0.04	0.98	0.07	0.02	1.00	7.30 × 10^−6^
18	rs146374374	intergenic	*CDH7* (dist=255064), *CDH19* (dist=355685)	C/A	0.03	0.96	0.06	0.02	1.00	6.68 × 10^−6^

Chr = chromosome; AF = allele frequency; HWE = Hardy Weinberg equilibrium

a Based on human genome assembly GRCh38. dist, distance to closest gene in base pairs (in parenthesis).

b Imputation r^2^

c *p* value < 5 × 10^−6^ indicates suggestive association.

**Table 2.** Results of association analysis of AP subphenotypes with and without pain vs. controls.

Chr^a^	SNP ID^a^	Alleles (1/2)^a^	Frequency Allele2	r^2^	BETA	SE	*P* value^c^	Allele Frequency Cases	Allele Frequency Controls	Location^a^	
2	rs35523245	T/C	0.11	0.97	1.13	0.22	3.89 × 10^−7^	0.16	0.09	intronic	*THADA*
2	rs6706102	G/A	0.11	1.00	0.96	0.21	4.31 × 10^−6^	0.16	0.08	intergenic	*MIR4268* (dist=390354), *EPHA4* (dist=1121108)
3	rs61530423	T/C	0.02	0.99	2.25	0.48	2.55 × 10^−6^	0.05	0.00	intronic	*GRM7*
5	rs955077	T/C	0.50	0.97	−0.67	0.13	1.22 × 10^−7^	0.40	0.55	intergenic	*IRX1* (dist=132655), *LINC02114* (dist=1039423)
5	rs4976592	T/C	0.71	0.95	0.70	0.15	1.79 × 10^−6^	0.78	0.67	intronic	*WWC1*
6	rs185577144	C/T	0.01	0.92	3.22	0.64	5.59 × 10^−7^	0.03	0.00	intronic	*MAK*
7	rs62454294	A/G	0.24	0.94	0.70	0.15	2.63 × 10^−6^	0.32	0.20	intergenic	*CYTH3* (dist=24222), *FAM220A* (dist=32565)
7	rs190613827	A/C	0.03	0.96	1.83	0.40	3.81 × 10^−6^	0.06	0.01	intergenic	*LOC389602* (dist=364638), *LOC285889* (dist=104189)
7	rs75624382	T/C	0.01	1.00	2.35	0.51	4.80 × 10^−6^	0.04	0.00	intronic	*CNTNAP2*
9	rs62532967	T/C	0.26	0.96	0.72	0.15	1.04 × 10^−6^	0.34	0.21	intergenic	*KDM4C* (dist=340779), *DMAC1* (dist=280073)
9	rs200642523	CT/C	0.74	0.92	−0.71	0.15	1.63 × 10^−6^	0.66	0.79	intronic	*BNC2*
12	rs959346	G/A	0.49	0.95	0.71	0.14	2.87 × 10^−7^	0.56	0.45	intronic	*SLCO1C1*
13	rs34063893	G/A	0.02	0.96	2.39	0.52	4.49 × 10^−6^	0.04	0.00	intergenic	*ING1* (dist=4414), *LINC00567* (dist=81925)
14	rs149775990	A/G	0.02	0.95	2.41	0.52	3.87 × 10^−6^	0.04	0.01	intronic	*SYT16*
15	rs77314700	G/A	0.01	0.97	2.57	0.54	1.93 × 10^−6^	0.04	0.00	intergenic	*RNU6–9* (dist=4914), *PIAS1* (dist=209356)

AP subphenotypes were based on the presence [acute apical abscess (AAA), symptomatic apical periodontitis (SAP)] or absence [chronic apical abscess (CAA), asymptomatic apical periodontitis (AAP)] of pain based on the AAE’s diagnostic criteria.

a Based on human genome assembly GRCh37. dist, distance to closest gene in base pairs (in parenthesis).

b Imputation r^2^

c *p* value < 5 × 10^−6^ indicates suggestive association.

**Table 3.** Results of sex-specific single-variant association analysis.

Sex	Chr.	SNP ID^a^	Location	Gene/locus	Alleles (1/2)	Allele 2 frequency	Allele frequency Cases	Allele frequency Controls	*P* value^b^
Males (n=373)	1	rs2905035	ncRNA	*LINC01128*	A/G	0.74	0.65	0.79	**1.34 × 10** ^**−6**^
	1	rs142486420	intronic	*C1orf21*	C/T	0.02	0.04	0.00	**3.05 × 10** ^**−6**^
	5	rs4700466	intergenic	*C5orf64-AS1, KIF2A*	T/C	0.58	0.47	0.65	**1.19 × 10** ^**−7**^
	6	rs114963604	intronic	*TAF8*	G/A	0.01	0.04	0.00	**2.32 × 10** ^**−6**^
	7	rs6467700	intergenic	*LOC349160, PTN*	G/A	0.45	0.56	0.38	**2.45 × 10** ^**−6**^
	9	rs78788856	intronic	*COL15A1*	G/C	0.03	0.07	0.01	**1.12 × 10** ^**−6**^
	12	rs73105376	intergenic	*LINC02156, RPAP3*	G/A	0.02	0.05	0.00	**6.65 × 10** ^**−7**^
	13	rs9634708	intergenic	*RFC3 LINC02343*	T/C	0.06	0.11	0.03	**3.58 × 10** ^**−6**^
	14	rs2766688	intergenic	*DEGS2, YY1*	A/G	0.02	0.04	0.00	**2.23 × 10** ^**−6**^
	19	rs77632739	intronic	*AXL*	C/T	0.01	0.03	0.00	**4.86 × 10** ^**−6**^
	20	rs13038344	intronic	*RALGAPB*	A/G	0.08	0.14	0.04	**1.52 × 10** ^**−7**^
Females (n=506)	2	rs1187362395	intronic	*CREG2*	C/T	0.03	0.05	0.02	**3.65 × 10** ^**−6**^
	2	rs75047629	intronic	*CNTNAP5*	A/G	0.01	0.03	0.00	**3.73 × 10** ^**−6**^
	3	rs62247663	intergenic	*DAZL, PLCL2*	G/T	0.23	0.14	0.28	**2.72 × 10** ^**−6**^
	4	rs117436459	intergenic	*LINC02429, MIR548AG1*	G/T	0.03	0.05	0.01	**1.50 × 10** ^**−6**^
	4	rs6844234	intergenic	*PDHA2, LINC02267*	T/C	0.33	0.21	0.40	**7.32 × 10** ^**−7**^
	6	rs147072993	intergenic	*EDN1, LINC02530*	C/G	0.01	0.03	0.00	**3.95 × 10** ^**−6**^
	8	rs7835237	intergenic	*STC ADAM28*	C/G	0.03	0.06	0.01	**4.18 × 10** ^**−6**^
	11	rs1863633841	intergenic	*TRPC6, ANGPTL5*	G/T	0.07	0.09	0.06	**4.03 × 10** ^**−6**^
	12	rs3832832	exonic	*NCKAP1L*	G/A	0.03	0.02	0.04	**4.95 × 10** ^**−6**^
	13	rs678622	intronic	*SGCG*	G/A	0.59	0.71	0.52	**2.69 × 10** ^**−6**^
	15	rs61045542	ncRNA	*PLA2G4E-AS1*	G/A	0.10	0.15	0.07	**2.55 × 10** ^**−6**^
	16	rs222908	intronic	*MPV17L*	T/C	0.55	0.48	0.60	**9.24 × 10** ^**−7**^
	18	rs1452590	intergenic	*CDH20, LINC01544*	A/C	0.71	0.61	0.77	**1.21 × 10** ^**−6**^
	19	rs56391275	Insertion/deletion	*DMKN*	ACCACTGCTAT/A	0.16	0.22	0.13	**3.79 × 10** ^**−6**^
	19	rs8105480	intronic	*MYH14*	T/C	0.07	0.03	0.09	**2.56 × 10** ^**−6**^

a Based on human genome assembly GRCh37.

b *p* value < 5 × 10^−6^ indicates suggestive association (bold)

## Data Availability

The datasets generated during and/or analyzed during the current study are available in dbGaP repository.
